# Depression and Its Determinants among Postpartum Mothers Attending at Universal College of Medical Sciences and Teaching Hospital, Bhairahawa, Rupandehi, Nepal

**DOI:** 10.1155/2023/1331641

**Published:** 2023-07-26

**Authors:** Chet Kant Bhusal, Sigma Bhattarai, Alisha Shrestha, Hem Raj Sharma

**Affiliations:** ^1^Department of Community Medicine, Universal College of Medical Sciences and Teaching Hospital Affiliated to Tribhuvan University, Bhairahawa, Rupandehi, Nepal; ^2^Department of Nursing, Universal College of Medical Sciences and Teaching Hospital Affiliated to Tribhuvan University, Bhairahawa, Rupandehi, Nepal; ^3^Atoot, Kalanki 14, Kathmandu, Nepal; ^4^Rapti Provincial Hospital Tulsipur Dang, Nepal

## Abstract

**Background:**

Postpartum depression is a serious mental health issue linked to maternal morbidity and negative effects for infant's normal growth, development, and well-being. This study is aimed at assessing the prevalence and factors associated with postpartum depression among mothers attending a tertiary hospital in Nepal.

**Methods:**

A hospital-based cross-sectional study was conducted among 173 postpartum mothers (<6 weeks postdelivery) who were receiving care either at the postnatal ward or immunization clinic of Universal College of Medical Sciences and Teaching Hospital in Bhairahawa, Rupandehi district, Nepal. The study was carried out from October 2020 to February 2021 by using purposive sampling technique for selecting respondents. The variables that showed significant association with the dependent variable having *p* value < 0.05 in bivariate analysis were entered into multivariate logistic regression model to find the final associated factors.

**Results:**

The prevalence of postpartum depression was 20.2% among mothers attending a tertiary hospital in Nepal. The mean age of the respondents was 24.77 ± 3.47. Factors such as mothers having female child (AOR = 6.39, CI = 1.54 − 26.46), unplanned pregnancy (AOR = 10.08, CI = 2.91 − 34.94), pregnancy-induced health problems (AOR = 9.68, CI: 3.51-26.64) were associated with an increased risk of postpartum depression. Similarly, mothers having formal education (AOR = 0.28, CI: 0.08-0.91), whose spouses have secondary and above education (AOR = 0.16, CI: 0.03-0.85), and who have ≥4 ANC visits (AOR = 0.15, CI = 0.05 − 0.40) were significantly associated but have a protective effect with postpartum depression.

**Conclusions:**

Sex of newborn, mother's and spouse's education, intention of pregnancy, ANC visits, and pregnancy-induced health problems were found to be significantly associated with postpartum depression. Hence, specific health education program regarding maternal and child health integrating mental health should be provided to pregnant women, mothers, and their husbands, focusing on gender discrimination. Similarly specific orientation program should be provided to local health worker about the importance of planned pregnancy, in order to reduce pregnancy related health problems during ANC visits and to mothers after their delivery to reduce further chances of postpartum depression.

## 1. Introduction

Depression is the leading cause of disability, accounting for a significant portion of the worldwide disease burden and impacting more women than men [1]. Maternal mental health problems are a major public health issues all over the world [2]. Pregnancy and postpartum periods are particularly vulnerable periods for every woman. As a result, women are at a higher risk of acquiring mental health issues, especially depression and anxiety, since they go through so many bodily and emotional changes during this time [3].

Depression during the postpartum period is one of the most common psychosocial consequences of motherhood [4]. When postpartum depression is left untreated, it can impact not only the mother's health and quality of life but also the infant's normal growth, development, and well-being [4]. Postpartum depression (PPD) has been associated to a number of fatal outcomes, including infanticide and mother suicide [5, 6]. It also has a negative impact on the child's growth and development [7, 8]. Child neglect and abandonment are likely outcomes of depressive symptoms during the postpartum period [9]. In comparison to children of nondepressive mothers, children of postpartum depressed mothers had much more cognitive, behavioral, and interpersonal difficulties [10, 11]. Despite, the fact that postpartum depression is harmful to both the mother and her child, it is still underdiagnosed and consequently neglected. Women with postpartum depression are still unwilling to seek professional care because of the stigma associated with mental illness in society [4], their inability to recognize it as a depressive condition or are unaware of the symptoms, and a lack of appropriate health education offered to them throughout their pregnancy [12]. If postpartum depression gets worse, it can lead to more serious conditions such as psychosis and possibly death [13]. Postpartum depression is a serious mental health issue which is linked to maternal morbidity and negative effects for the baby. The first six months following birth are a particularly vulnerable period for developing depression [14].

Postpartum depression affects 13% to 19% of pregnant women, making it one of the most common consequences of delivery [15, 16]. In low- and middle-income countries, postpartum depression is the most frequent mental health condition among women of reproductive age [17]. In such countries, postpartum depression affects 20% to 40% of women [18]. A systematic review conducted in low- and middle-income countries found that the prevalence of postnatal depression varies significantly based on the study setting, affecting roughly 20% of women in low-income countries [19]. Several studies conducted in different parts of Nepal found postpartum depression as a major problem [9, 13, 20, 21].

During the postnatal follow-up, mothers are not properly screened for postpartum depression, and no cases of postpartum depression are referred to appropriate mental health treatments [22]. Referral to mental health services for depression treatment is a complex topic with a number of interconnected layers within the health care systems [22]. Postpartum depression is a serious but manageable medical illness that manifests in a variety of symptoms; thus, it requires appropriate attention and treatment to improve both the maternal health and health of the baby. However, there is a scarcity of data regarding effective psychological treatments [23]. In Nepal, postpartum depression is common, but it is an insufficiently researched component of maternal health that requires early-stage identification [24]. Hence, the objective of this study is to assess the prevalence and factors associated with postpartum depression among mothers attending a tertiary hospital in Nepal.

## 2. Materials and Methods

### 2.1. Study Design and Source of Population

A hospital-based cross-sectional study was conducted among postpartum mothers who had given birth within six weeks at Universal College of Medical Sciences and Teaching Hospital, Bhairahawa, Rupandehi, Nepal. The study was conducted from October 2020 to February 2021. Mothers attending the postnatal ward and immunization clinic of the hospital within six weeks of giving birth were included in the study, whereas mothers who were in severe pain due to delivery, who had severe mental health problems previously, and who refused to give consent and responses were excluded from the study.

### 2.2. Sample Size Determination and Sampling Technique

The sample size was calculated by using the formula *n* = *Z*^2^ pq/*L*^2^ with a 95% confidence interval, a critical value of *Z* = 1.96, and a 5% margin of error, and the prevalence of postpartum depression was taken as 12.27% [25]. Hence, *n* = *Z*^2^ pq/*L*^2^ = (1.96)^2^ × (0.1227) × (0.8773)/(0.05)^2^ = 165. Further, a 5% nonresponse rate was added to the initial sample. Therefore, a final sample size of the study was 173. Those mothers within six weeks of delivery attending either the postnatal ward or immunization clinic of the selected hospital were initially assessed to determine their eligibility based on the inclusion criteria, and then they were selected purposively after taking written informed consent.

### 2.3. Data Collection Procedure and Validity

Questionnaire regarding sociodemographic, socioeconomic, maternal, and obstetrical-related factors was formulated by reviewing different literatures and was translated into Nepali language and retranslated into English to find the misinterpretation, and necessary correction was made. For screening of postpartum depression, a validated Nepali version Edinburgh postnatal depression scale (EPDS) was used. The EDPS tool has been validated in different settings and languages and is also used in Nepal [9, 20, 26]. This tool has a sensitivity of 92% and a specificity of 95.6% at the cut-off point of ≥13 [7]. Direct face-to-face interview was used as data collection techniques.

### 2.4. Data Management and Analysis

Collected data was checked, coded, and edited manually by the researcher each day to ensure its consistency, completeness, and accuracy. Data was entered into Microsoft Excel and was analyzed by using the database IBM Statistical Package for the Social Sciences (SPSS) version 25. Initially, descriptive statistics such as frequency, percentages, mean, and standard deviation was calculated. Bivariate analysis was used to generate a logistic regression analysis to reveal the association between postpartum depression in women within six weeks of delivery with various sociodemographic, socioeconomic, maternal, and obstetrical-related factors. After that, all the variables that showed significant association with the dependent variable having *p* value < 0.05 in bivariate analysis were entered into the final multivariate logistic regression model to find the final associated factors. The Nagelkerke R square and the variation inflation factor (VIF) were used to assess the goodness of fit, and the multicollinearity of the variables was checked. The Nagelkerke R square value was 0.461, which indicates adequate goodness of fit. Similarly, VIF for all independent variables was less than 10, ranging from 1.05 to 1.29; hence, multicollinearity was not present among independent variables. After testing all of this, the multivariate logistic regression was conducted to identify the independent factors associated with postpartum depression.

### 2.5. Measurement of Postpartum Depression

The Edinburgh postnatal depression scale (EPDS), a useful and effective tool having 10 questions, was used for screening and identifying postpartum depression. Responses were scored 0, 1, 2, or 3 according to the increased severity of the symptom. The total score was determined by adding together the scores for each of the 10 items. Respondents who had EPDS score greater than or equal to 13 were considered as having postpartum depression, whereas those who had lesser than 13 scores were considered as having no depression as per the previous study [13].

### 2.6. Ethical Consideration and Consent to Participate

The ethical approval was obtained from the Institutional Review Committee of the Universal College of Medical Sciences and Teaching Hospital (UCMS/IRC/115/20) for this study. After sharing the study details, written informed consent was obtained from the respondents. Mothers who were illiterate had their thumbprints obtained, while mothers under the age of 18 years provided both their assent and their parents' informed consent. Each of the respondents was informed that their privacy and confidentiality would be protected to the fullest extent possible. Respondents were informed that the information gathered would only be used for research purposes.

## 3. Results

Among the 173 respondents, more than half of the respondents were from the age group 16–25 years with a mean age of 24.77 and a standard deviation of 3.47. More than three-fourths (75.7% and 76.9%) lived in a joint family and had more than four family members in the family, respectively. Majority of the respondents gave birth to their child within seven days (91.3%) and had a birth space of more than two years (65.5%). More than one-fifth of the respondents did not have formal classes (8.7% were illiterate, and 12.1% could read and write their names only), and 32.9% had an education level up to SLC or above. Less than half (47.4%) of the respondent's husband had attained an education level of SLC or above. Majority (91.3%) of the respondents were housewives, and more than one-fourth (26.0%) of the respondent's husbands were involved in labour. More than four-fifths (86.1%) of the respondents had their monthly family income of more than NRs. 20000 (Table 1).

The findings revealed that among the total respondents, 50.3% had a female child. More than four-fifths (84.4%) of the respondents had cesarean section as a mode of delivery. More than half (50.3%) had two or more children, and more than four-fifths (87.3%) had their planned pregnancy. About three-fourths of the respondents had gone for four antenatal checkups (74.6%) while 94.2% of them had less than three postnatal checkups. Among the respondents, 76.3% did not report any pregnancy-related health problems (Table 2).

Table 3 represents the prevalence of postpartum depression among mothers attending UCMS, Bhairahawa. About one-fifth (20.2%) of the respondents had postpartum depression when assessed through Edinburgh postnatal depression scale (EPDS score ≥ 13) (Table 3).

Sociodemographic and socioeconomic characteristics related to depression among postpartum mothers were shown in Table 4. After adjusting to multivariate regression analysis model variables such as sex of the newborn, mother's and spouse's education were found to be associated with postpartum depression. Mothers having female child were 6.39 times more likely (AOR = 6.39, CI = 1.54 − 26.46) to be depressed during their postpartum period. The odds of having postpartum depression were 0.28 times less likely (AOR = 0.28, CI: 0.08-0.91) among those who have formal education as compared to those who were illiterate. Similarly, mothers whose spouses had secondary or higher education were found to be 0.16 times less likely to experience postpartum depression (AOR = 0.16, CI: 0.03-0.85) compared to those whose spouses had primary or informal education (Table 4). Several variables such as the age of the mother, type of family, size of family, age of index child, occupation of the mother, and occupation of the father were not found to be significantly associated with postpartum depression.

Table 5 represents maternal- and obstetrical-related factors associated with postpartum depression among mothers attending Universal College of Medical Sciences, Bhairahawa, Rupandehi, Nepal. After adjusting to multivariate regression analysis model variables such as intent of pregnancy, ANC visits and pregnancy-induced health problems were found to be associated with postpartum depression. Mothers who did not plan their pregnancy were 10 times more likely (AOR = 10.08, CI = 2.91 − 34.94) to have depression in their postpartum period. The odds of having postpartum depression were reduced by 15% (AOR = 0.15, CI = 0.05 − 0.40) among mothers who had their ANC visits for more than equal to four times. Similarly, the odds of having postpartum depression were 9.68 times more likely (AOR = 9.68, CI: 3.51-26.64) among those who have pregnancy-induced health problems. After being subjected to a multivariate model, PNC visit was not found to be significantly associated with postpartum depression (Table 5). Variables such as mode of delivery and parity were not found to be significantly associated with postpartum depression.

## 4. Discussion

Variables such as sex of newborn, mother's and spouse's education, intent of pregnancy, ANC visit, and pregnancy-induced health problems were independently associated factors of having depression among postpartum mother in this study. Mothers having female child were found to be more depressed during their postpartum period; this might be due to male-dominant Nepalese society. Postpartum depression was found to be more in those mothers who did not plan their pregnancies. The finding of this study revealed that one-fifth of the mothers have depression during their postpartum period which is consistent with the findings of the several other studies, such as studies conducted in Nepal [20, 27–30], Danang City, Vietnam [31], Central Java, Indonesia [32], and Nekemte town, west Ethiopia [33]. However several other studies such as study done in Paropakar Maternity and Women's Hospital, Nepal [9], Dhulikhel Hospital of Kavre district Nepal [34], Bharatpur metropolitan city Chitwan Nepal [5], and Southwest, Ethiopia [35] found higher proportion of the postpartum depression as compared to the current study. In contrast to this study, several other studies such as study conducted in Tertiary Health Care, Nepal [24], Pokhara Nepal [21], Janaki Medical College and Teaching Hospital, Dhanusha, Nepal [13], and Morang, Jhapa and Sunsari districts of Nepal [36], Iran [37] revealed lower prevalence of postpartum depression as compared to the present study. This variation in prevalence rate might be due to the variations in methodological design, length of the postpartum period, the population characteristics, and use of different cut-off points of EPDS score.

Around half proportion of the mothers in this study were primiparous which is in line with another study conducted in Kadaghari Kathmandu, Nepal [38], Parsa District of province number 2 of Nepal [17]. Less than one-fifth of the mothers in the present study had planned their pregnancy; however, in contrast to this study, several other studies such as a study conducted at Parsa [17] and Dhanusha district of Nepal [9] found that more than half of the mothers had planned their pregnancy. The observed differences in these studies might be due to the difference in sample size, educational and employment opportunities, and use of varying cut-off points of EPDS scores.

In the current study, postpartum depression was found more among mothers who were illiterate and have informal education in comparison to their counterparts which is in line with several other studies such as a study conducted in Bharatpur city of the central-southern part of Nepal [5], eastern Nepal [39], Saudi Arabia [40], and upper Egypt [41]. Spouse education was found significantly associated with having postpartum depression. This finding was supported by the study conducted in Egypt [41]. This study revealed that having a female child has a significant association with postpartum depression where mothers having female child were more likely of having depression compared to those having a male child. This finding was in consistency with a study conducted in Mandya district, Karnataka state, India [42]. In the present study, there is a significant association between the intent of pregnancy and postpartum depression where the odds of developing depression were more likely for unplanned pregnancy. This finding aligns with the studies conducted in Lalitpur, Nepal [27], Parsa district of the southern plains of Nepal [17], Bangladesh [43], Southwest Ethiopia [35], and Eritrea [18]. In this study, postpartum depression was significantly associated with number of times of antenatal checkup visits during their pregnancy period revealing the odds of occurrence of postpartum depression to be lower in mothers completing recommended 4 or more antenatal checkups, which is in accordance with a study conducted in southern Nepal [17] and Srilanka [44]. This study revealed that having pregnancy-induced health problems increased the likelihood of being depressed in the postpartum period which is in accordance with other studies, such as a study conducted in Paropakar Maternity and Women's Hospital, Kathmandu Nepal [9], Lalitpur district, Nepal [27], India [42], and Eritrea [18].

In the present study, age of the mothers was not found statistically significant with having postpartum depression which is in line with another study conducted at Dhanusha district of Nepal [9]; however, in contrast to this study, several other studies such as the study conducted at Kathmandu Nepal [9], Srilanka [44], and Southwest Ethiopia [35] found that the age of mothers was significantly associated with having postpartum depression. This divergence might be attributed to the increase in educational and employment opportunities, particularly among the youth in previous studies. The parity in this study was not found statistically significant with having postpartum depression; however, in contrast to this study, another study conducted at Srilanka [44] found that parity was significantly associated with having postpartum depression. Similarly, in the present study, age of the index child was not significantly associated with postpartum depression. This finding was inconsistent with a study conducted in Uganda [45]. The variation observed might be attributed to differences in data collection techniques, measurement tools, and the distinct social and cultural characteristics across various countries.

## 5. Conclusions

One-fifth of the mothers in the present study were found to have postpartum depression. Mother's and spouse's education, sex of newborn, intention of pregnancy, ANC visits, and pregnancy-induced health problems were found to be significantly associated with postpartum depression. Health planner and policymakers should focus on specific health education program regarding maternal and child health integrating mental health for the pregnant women, mothers, and their husbands, focusing on gender discrimination. Local health care workers were recommended to provide counseling on maternal mental health, especially postpartum depression and addressed pregnancy-related health problems to the pregnant women during ANC visits and to mothers after their delivery to reduce further chances of depression during the postpartum period. Early screening procedures in health care services should be performed for early diagnosis of depressive symptoms.

### 5.1. Limitations

The nonprobability purposive sampling method and the use of the EPDS tool for screening depressed symptoms without a clinical diagnosis are two major limitations of the study. Since the study was cross-sectional and only included a minimal number of postpartum women in its sample, it is recommended to undertake further analytical and interventional studies to determine the causal relationship between the linked factors.

## Figures and Tables

**Table 1:** Distribution of sociodemographic and socioeconomic characteristics of study population.

Characteristics	Frequency (*n* = 173)	Percentage
Age of mothers		
16–25 years	104	60.1
26–35 years	69	39.9
Mean ± standard deviation	24.77 ± 3.47	
Type of family		
Nuclear	42	24.3
Joint and extended	131	75.7
Size of family		
≤4 members	40	23.1
>4 members	133	76.9
Age of index child		
≤7 days	158	91.3
>7 days	15	8.7
Sex of newborn		
Male	87	50.3
Female	86	49.7
Birth spacing of index child (*n* = 87)		
≤2 years	57	65.5
>2 years	30	34.5
Education of mother		
Illiterate	15	8.7
Literate	21	12.1
Primary level	23	13.3
Lower secondary level	29	16.8
Secondary level	28	16.2
SLC or above	57	32.9
Education of father		
Illiterate	3	1.7
Literate	2	1.2
Primary level	11	6.4
Lower secondary level	31	17.9
Secondary level	44	25.4
SLC or above	82	47.4
Occupation of mother		
Housewife	158	91.3
Farmer	3	1.7
Service	8	4.6
Business/self-employed	3	1.7
Labour	1	0.6
Occupation of father		
Farmer	44	25.4
Service	38	22.0
Business/self-employed	26	15.0
Labour	45	26.0
Foreign employment	9	5.2
Unemployed	11	6.4
Monthly family income (Nepalese rupees NRs)		
≤NRs. 20000	24	13.9
>NRs. 20000	149	86.1

SLC: school leaving certificate.

**Table 2:** Distribution of maternal and obstetrical related characteristics of the respondents.

Characteristics	Frequency (*n* = 173)	Percentage
Mode of delivery		
Cesarean section	146	84.4
Vaginal delivery	27	15.6
Parity		
Primiparous	86	49.7
Multiparous	87	50.3
Intent of pregnancy		
Planned	23	13.3
Unplanned	150	86.7
ANC visits		
<4 visits	129	25.4
≥4 visits	44	74.6
PNC visits		
<3 visits	163	94.2
≥3 visits	10	5.8
Pregnancy-induced health problems		
Yes	41	23.7
No	132	76.3

**Table 3:** Prevalence of postpartum depression among the study population.

Postpartum depression	Frequency (*n* = 173)	Percentage
Depression (EPDS score ≥ 13)	35	20.2
No depression (EPDS score < 13)	138	79.8

**Table 4:** Sociodemographic and socioeconomic factors associated with postpartum depression on using bivariate and multivariate analysis.

Characteristics	Postpartum depression		*p* value	^a^COR 95% CI	^b^AOR 95% CI
	Yes (EPDS ≥ 13) *n* (%)	No (EPDS < 13) *n* (%)			
Sex of newborn					
Male	29 (33.3)	58 (66.7)	<0.001^∗^	1	1
Female	6 (7.0)	80 (93.0)		6.67 (2.60-17.09)	6.39 (1.54-26.46)
Birth space of index child (*n* = 87)					
≤2 years	8 (14.0)	49 (86.0)	0.006^∗^	1	1
>2 years	12 (40.0)	18 (60.0)		0.25 (0.09-0.70)	0.48 (0.14-1.70)
Mother's education					
Illiterate and informal education	15 (41.7)	21 (58.3)	0.001^∗^	1	1
Formal education	20 (14.6)	117 (85.4)		0.24 (0.11-0.54)	0.28 (0.08-0.91)
Spouse's education					
Informal and primary	9 (56.3)	7 (43.7)	<0.001^∗^	1	1
Secondary and above	26 (16.6)	131 (83.4)		0.15 (0.05-0.45)	0.16 (0.03-0.85)

^∗^Significant at *p* < 0.05; 1 = reference category; ^a^crude odds ratio; ^b^adjusted odds ratio.

**Table 5:** Maternal and obstetrical factors associated with postpartum depression on using bivariate and multivariate analysis.

Characteristics	Postpartum depression		*p* value	^a^COR 95% CI	^b^AOR 95% CI
	Yes (EPDS ≥ 13) *n* (%)	No (EPDS < 13) *n* (%)			
Intent of pregnancy					
Planned	13 (56.5)	10 (43.5)	<0.001^∗^	1	1
Unplanned	22 (14.7)	128 (85.3)		7.56 (2.95-19.37)	10.08 (2.91-34.94)
ANC visits					
<4 visits	13 (10.1)	116 (89.9)	<0.001^∗^	1	1
≥4 visits	22 (50.0)	22 (50.0)		0.11 (0.05-0.26)	0.15 (0.05-0.40)
PNC visits					
<3 visits	30 (18.4)	133 (81.6)	0.016^∗^	1	1
≥3 visits	5 (50.0)	5 (50.0)		0.23 (0.06-0.83)	0.45 (0.09-2.28)
Pregnancy-induced health problems					
No	23 (56.1)	18 (43.9)	<0.001^∗^	1	1
Yes	12 (9.1)	120 (90.9)		12.77 (5.42-30.07)	9.68 (3.51-26.64)

^∗^Significant at *p* < 0.05; 1 = reference category; ^a^crude odds ratio; ^b^adjusted odds ratio.

## Data Availability

The raw data under the identification policy will be provided, upon request through email to the corresponding author.

## Abbreviations

ANC: Antenatal care
AOR: Adjusted odds ratio
CBS: Central Bureau of Statistics
CI: Confidence interval
EPDS: Edinburgh postnatal depression scale
IRC: Institutional review committee
NMC: Nepal Medical Council
NRS: Nepalese rupees
OR: Odds ratio
PNC: Postnatal care
PPD: Postpartum depression
SD: Standard deviation
SLC: School leaving certificate
SPSS: Statistical package for social sciences
UCMS-TH: Universal College of Medical Sciences and Teaching Hospital
VIF: Variation inflation factor.
